# Disagreement between mothers' and fathers' rating of health-related quality of life in children with cancer

**DOI:** 10.1007/s11136-023-03341-0

**Published:** 2023-01-12

**Authors:** Andreas Meryk, Gabriele Kropshofer, Benjamin Hetzer, David Riedl, Jens Lehmann, Gerhard Rumpold, Alexandra Haid, Verena Schneeberger-Carta, Christina Salvador, Evelyn Rabensteiner, Maria-Sophie Rothmund, Bernhard Holzner, Roman Crazzolara

**Affiliations:** 1 Department of Pediatrics, Medical University of Innsbruck, Anichstrasse 35, 6020 Innsbruck, Austria; 2 Department of Psychiatry, Psychotherapy and Psychosomatics, University Hospital of Medical Psychology, Medical University of Innsbruck, Innsbruck, Austria; 3 Department of Psychiatry, Psychotherapy and Psychosomatics, University Hospital of Psychiatry II, Medical University of Innsbruck, Innsbruck, Austria; 4 Department of Psychology, University of Innsbruck, Innsbruck, Austria; 5 Ludwig Boltzmann, Institute for Rehabilitation Research, Vienna, Austria

**Keywords:** Patient-reported outcome measure, Childhood cancer, HRQOL, Proxy, Parents, ePROtect

## Abstract

**Purpose:**

Serial assessment of health condition based on self-report made by children and their proxies has consistently shown a lack of congruence. The study explored the discrepancies between mother’s, father’s, and children’s reports on health-related quality of life (HRQOL) during the first two months of pediatric cancer treatment.

**Methods:**

In this cohort study, children and parents completed the generic and cancer-specific Pediatric Quality-of-Life Inventory (PedsQL) questionnaires at initial diagnosis and in the subsequent months. Evaluation of discrepancies included intraclass correlations between mother–child and father–child dyads at different domain levels.

**Results:**

Thirty-six children with a diagnosis of cancer between May 2020 and November 2021 and their parents were included in this study. At diagnosis, mother–child dyads showed better agreement on more domains of the PedsQL Generic Core Scale than father–child dyads; moderate agreement persisted for both parents at subsequent time points on the physical domain. The disease-specific PedsQL Cancer Module revealed moderate and better agreement for mother–child dyads during active cancer therapy. In particular, agreement of mother–child dyads was pronounced for domains such as worry (0.77 [95% CI 0.52–0.89, *P* < 0.001]), whereas fathers tended to overestimate the child’s symptom burden for most of the remaining domains of the PedsQL Cancer Module.

**Conclusion:**

This cohort study shows that both parent proxy reports can provide valid information on child’s HRQOL, but that fathers tend to overestimate, particularly for non-observable domains. Proxy reports derived from mothers more closely agreed with children’s HRQOL and might be more weighted, if there is uncertainty between parents.

**Supplementary Information:**

The online version contains supplementary material available at 10.1007/s11136-023-03341-0.

## Introduction

Overall survival for pediatric cancer has increased dramatically, resulting in more than 94% of patients surviving acute lymphoblastic leukemia [1, 2]. As a consequence, improvements in childhood cancer care have directed attention to the entire and complex situation in which the patients and their families find themselves. Acute and chronic health conditions such as pain, fatigue, nausea, anxiety, and depression commonly develop and ultimately pose a risk for social and economic challenges that reduce quality of life during and after completion of therapy [3, 4]. Thus, a central task in clinical care is to take these aspects seriously and to shift the focus also to the emotional and psychological stress.

The gold standard for assessing patients’ experience and health-related quality of life (HRQOL) is patient-reported outcome measurements (PROMs) [5–10]. While they are widely used in adult oncology, PROMs are still rarely present in pediatric cancer research and therapy [11]. The use of PROMs in this vulnerable group is compromised by age, developmental stage, family relationships and psychosocial challenges [12–14], but children can reliably self-report on their health if adequate questionnaires are used [15–17].

The health-care relationship in pediatrics is a triad, where the caregivers are involved in the management of the patient’s health conditions [18, 19]. Thus, caregiver proxy reports are often used as an alternative to the child’s self-report, particularly but not only when the child is unable to provide a self-report. The congruence between caregiver reports and child self-reports is influenced by several factors such as diagnosis, age, gender, socioeconomic status, or parent’s own HRQOL [20–23] with the consequence that disagreement on health and well-being might result in medical mismanagement [24]. In addition, parents consistently tend to overestimate symptom burden and functional limitations compared to children’s self-report [20, 22, 23, 25, 26]. Since an overall lack of fathers’ reports is noted [12], most of the studies compared caregiver–child dyads and there are little data regarding the difference between mothers’ and fathers’ perspective on child’s HRQOL.

One recently published study obtained proxy ratings from both parents separately and suggested that paternal and maternal reports are interchangeable. However, the majority of questionnaires reported results at 3.3 years after end of treatment and, thus, were not representing the impact of acute cancer therapy [27]. In general, treatment for childhood cancer can extent over several months, but the first 2–3 months of treatment represent the most intense phase, as they are associated with urgent surgery (e.g., tumor removal, insertion of catheters), highest toxicity by chemotherapy or irradiation, and residual cancer impairment (e.g., fatigue, pain). Together with the isolation from the usual social setting, this puts the patients in the focus of major interest to compare mothers’ and fathers’ perspective on children’s HRQOL.

We recently developed a unique, web-based approach for daily child self- and parent-based proxy reporting (ePROtect) [28–30]. Within this study, the child as well as both parents were asked to complete additionally the general and the cancer-specific version of the Pediatric Quality-of-Life Inventory (PedsQL) on a monthly basis. We aimed to compare cross-sectional as well as longitudinal agreement on HRQOL between children and adolescents newly diagnosed with cancer and their corresponding mothers and fathers in the first months of therapy.

## Patients and methods

### Participants

Only German-speaking children and adolescents with cancer who were age 5 to 18 years at enrollment, and their parents (legal guardians) were recruited for the study. Families with single parents were allowed to participate. Inclusion was not restricted to biological parents and also same-sex parents were eligible; however, both do not apply to this study. Start of chemotherapy within 15 days of diagnosis and a sufficient ability to fluently speak and understand German were inclusion criteria. Exclusion criteria were apparent cognitive disability or visual impairment that precluded utilization of the web application. The Ethics Committee of the Medical University of Innsbruck approved this study (EC Number: 1055/2020); written informed consent was obtained from all children and their parents. Socio-demographic and clinical data were collected at study inclusion.

### Study design

This study belongs to the “ePROtect project,” an observational cohort study started on May 01, 2020 at the pediatric oncology ward of the Medical University of Innsbruck (Austria). A detailed description of ePROtect has been published previously [28–30]. In addition to daily symptom monitoring performed by the children, patients and parents were instructed to complete PedsQL 4.0 Generic Core Scales and PedsQL 3.0 Cancer Module within seven days after diagnosis (T0) and then on a monthly basis for three months (T1, T2, T3). This time points represent important milestones of treatment. T0 corresponds to diagnosis and patients may suffer from cancer symptoms and fear of treatment, T1 is equal to remission induction and patients may have severe toxicity after first treatment, T2 and T3 are equal to consolidation and the children are already most of the time at home. All collected data were directly used in the clinical treatment of the patients. Data regarding daily symptom monitoring for identification of adverse events and support clinical management were previously published and are not part of this publication [28–30].

### Measurement tools and assessment

The age- and rater-specific versions of the PedsQL 4.0 Generic Core Scales, a multidimensional measure of general HRQOL, and the PedsQL 3.0 Cancer Module which focuses on the dimensions of health affected by pediatric cancer and its treatment, were used for the assessment of HRQOL. The PedsQL Generic contains 23 items forming four principal domains including physical functioning (8 items), emotional functioning (5 items), school functioning (5 items), and social functioning (5 items). The PedsQL Cancer comprises 27 items in eight subscales (level of pain (2), nausea (5), procedural anxiety (3), treatment anxiety (3), worry (3), cognitive problems (5), perceived physical appearance (3), and communication (3)). Children and families answered each item on a 5-point Likert scale, where 0 = no problem, 1 = almost never, 2 = sometimes, 3 = often, and 4 = almost always. Younger children (5 to 7 years) answered each item on a 3-point Likert scale adopting faces corresponding to frequencies: a smiley face for “0 = no problem,” a neutral face for “2 = sometimes,” and a frowning face for “4 = almost always.” Both questionnaires have a reference period of one month. With the PedsQL scoring, the average score for each item in the subscales of both the child self-reports and the proxy reports were calculated and then converted to a 0–100 scale, with higher scores indicating better HRQOL. The PedsQL was chosen as measure as it is the currently most frequently used questionnaires in clinical research [31], and it has been recommended to be used in longitudinal pediatric oncology studies [32].

### Outcome measurements

Our primary outcome was the agreement between child–mother dyads and child–father dyads for each subscale of the PedsQL 4.0 Generic and the PedsQL 3.0 Cancer Module. The secondary outcome was the analysis of the completion rate during the first three months after diagnosis of the child’s cancer.

### Statistical analysis

The data extraction date was January 31, 2022. Data were analyzed from extraction to April 15, 2022. Sample characteristics were calculated as absolute numbers, percentages, medians, and IQRs. Intraclass correlation coefficients (ICCs) were calculated between child self-reports and mother’s as well as father’s proxy reports, and their 95% CIs based on a two-way random effects model for absolute agreement. Intraclass correlation coefficients reference values: ICC < 0.5 = poor agreement, ICC between 0.5 and < 0.75 = moderate agreement, ICC between 0.75 and < 0.90 = good agreement, ICC > 0.90 = excellent agreement [33]. Differences were visualized as whisker plots, and paired t tests were applied. Differences were considered statistically significant at *p* < 0.05. All statistical analyses were performed using SPSS, version 26.0 (IBM Corporation). For data visualization, Prism, version 8.4 (GraphPad), was used.

## Results

### Patient and parent characteristics

Forty-three children and adolescents, who were first diagnosed with cancer between May 1, 2020 and November 30, 2021 were considered eligible for the study. Three children did not receive chemotherapy and three patients and/or parents were not able to understand German. After matching with the inclusion criteria, 37 individuals consented to participate but one patient lost interest in continuing the study and did not complete any questionnaire (Supplementary Fig. 1). Finally, thirty-six (97.3%) patients were included in this study. Patients had a median age of 10.7 (IQR, 6.9–13.5) years; eleven (30.6%) were female and 25 (69.4%) were male. The diagnoses included thirteen patients with acute leukemia (36.1%), eight patients with lymphoma (22.3%), five patients with central nervous system tumors (13.9%), four patients with soft-tissue sarcoma (11.1%), and six patients with other tumors (16.7%), including germ cell tumors (n = 4), Langerhans cell histiocytosis (n = 1), and neuroblastoma (n = 1). All patients received standard induction chemotherapy, including nine (25.0%) with surgery and five (13.9%) with both surgery and radiotherapy (Table 1).

**Table 1:** Demographic and clinical characteristics of the study cohort

Child characteristics	No. (%)
*Total* Female Male	36 11 (30.6) 25 (69.4)
Age, median (IQR), years	10.7 (6.9–13.5)
*Age group*	
5–7 years 8–12 years 13–18 years	13 (36.1) 14 (38.9) 9 (25.0)
*Underlying diagnosis*	
ALL AML Hodgkin’s lymphoma NHL CNS tumor STS Other	12 (33.3) 1 (2.8) 5 (13.9) 3 (8.4) 5 (13.9) 4 (11.1) 6 (16.7)
*Treatment*	
CTX CTX + surgery CTX + surgery + radiotherapy	22 (61.1) 9 (25.0) 5 (13.9)
Parent characteristics	No (%)
*Total*	59
Mothers Fathers	35 (59.3) 24 (40.7)
*Age, median (IQR), years*	
Mothers Fathers	43.5 (37.3–47.4) 46.6 (41.2–49.9)*
*Marital status*	
Married/living together Single/divorced/widowed	23 (63.9) 13 (36.1)
*Primary caregiver*	
Mother Father	35 (97.2) 1 (2.8)

ALL: acute lymphoblastic leukemia, AML: acute myeloblastic leukemia, NHL: non-Hodgkin’s lymphoma, CNS: central nervous system, STS: soft-tissue sarcoma, CTX: chemotherapy.

*Age of one father unknown

Of all 36 included patients, all legal guardians were approached and asked to take part in the study. All of them (100%) consented to participate, resulting in the inclusion of 35 (59.3%) mothers and 24 (40.7%) fathers. Median age of mothers was slightly but not significantly lower than that of fathers (43.5 years versus 46.6 years, *p* = 0.52). In 23 (63.9%) families, parents were married or lived together. Single parents were mostly mothers, except for one case. Data on hospital admission revealed that mothers spent most of their time during inpatient stay with the child and were the primary caregiver in nearly all cases (97.2%) (Table 1).

### Participation in PRO assessments

**Fig. 1:**
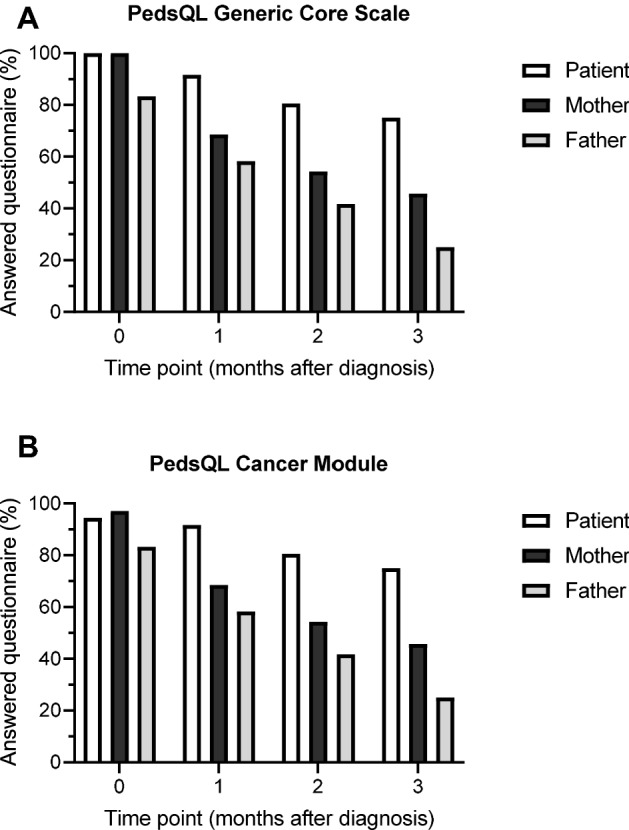
Completion of monthly provided questionnaires to measure child’s HRQOL. 36 children, 35 mothers and 24 fathers were approached to complete monthly Pediatric Quality-of-Life Inventory (PedsQL) 4.0 Generic Core Scales (A) and PedsQL 3.0 Cancer Module (B). Completion rate for patients (white), mothers (dark gray), and fathers (light gray) is shown for different time points. Time point 0 shows completion within seven days following diagnosis of cancer and the subsequent time points are on a monthly basis

At time of diagnosis, all children and mothers answered PedsQL Generic, but two patients and one mother did not complete the PedsQL Cancer (Fig. 1 A + B). Despite consent to participate, four out of 24 fathers did not complete the two proxy questionnaires, thus, giving a participation rate of 83% at time of diagnosis. With ongoing time, participation of mothers and fathers decreased more than that of children. Completion rates for fathers were the lowest at all time points and only six (25%) fathers participated three months after diagnosis compared to sixteen (46%) mothers. Due to the low participation rate for fathers at time point 3, this time point was not included in the analysis.

### Agreement between child and parents on PedsQL generic

Table 2 shows ICCs between child self-report and parent proxy report for the PedsQL Generic, stratified for mothers and fathers. At time of diagnosis, mother–child dyads showed moderate to good agreement on all domains, except for social functioning, whereas father–child dyads showed only moderate agreement on physical functioning and school domain. With ongoing therapy, moderate agreement remained only on the physical domain for both mother–child and father–child dyads at time point 1 and was present only for mother–child dyads at time point 2. Disagreement seemed to be linked to tendency of mothers and fathers to overestimate impairments (Supplementary Fig. 2).

**Table 2:** Agreement between parent and child dyads on PedsQL 4.0 Generic Core Scale

	n	Mother and child reports		n	Father and child reports	
		ICC	95% CI		ICC	95% CI
*Time point 0*						
Physical Emotional Social School Total	35	0.78*** 0.65*** 0.36* 0.52*** 0.80***	0.61–0.88 0.61–0.88 0.04–0.62 0.21–0.73 0.64–0.89	20	0.58** 0.45* 0.42* 0.52* 0.56**	0.18–0.81 0.02–0.74 0.00–0.72 0.09–0.78 0.15–0.80
*Time point 1*						
Physical Emotional Social School Total	23	0.68*** 0.13 0.39* 0.42* 0.51**	0.31–0.86 − 0.15–0.44 0.02–0.68 − 0.02–0.74 0.08–0.77	13	0.54* 0.39* 0.26 0.19 0.34	0.06–0.83 − 0.12–0.77 − 0.20–0.67 − 0.29–0.69 − 0.13–0.72
*Time point 2*						
Physical Emotional Social School Total	19	0.74*** 0.20 0.26 0.43 0.52***	0.22–0.91 − 0.16–0.55 − 0.11–0.60 − 0.02–0.75 − 0.02–0.80	10	0.40* 0.32 0.34 0.07 0.31	− 0.13–0.80 − 0.17–0.74 − 0.19–0.76 − 0.61–0.70 − 0.18–0.74

ICC Intraclass correlation coefficient (two-way mixed model. Absolute agreement, CI 95% confidence interval

*p ≤ 0.05; **p ≤ 0.01; ***p ≤ 0.001

Intraclass correlation coefficients reference values: ICC < 0.5: poor agreement, ICC between 0.5 and < 0.75: moderate agreement, ICC between 0.75 and < 0.90: good agreement, ICC > 0.90: excellent agreement [33]

Bold indicates significant values with at least moderate agreement

### Agreement between child and parents on PedsQL cancer

At time of diagnosis, mother–child and father–child dyads showed similar number of domains with moderate and good agreement for the PedsQL Cancer module (Table 3). One month after diagnosis, agreement in all domains was absent in father–child dyads, as stronger impairments were reported by fathers than from the child’s own perspective (Supplementary Fig. 3). In the case of mother–child dyads, moderate agreement was reported not only for the observable domains (e.g., pain and hurt, nausea) but also good agreement for the domain worry (0.77 [95% CI 0.52–0.89, *P* < 0.001]). At the last assessment, father–child dyads showed moderate agreement for the domains nausea (0.51 [95% CI − 0.05–0.84, *P* < 0.04]) and procedural anxiety (0.56 [95% CI − 0.00–0.86, *P* < 0.02]), which in turn exhibited excellent agreement for mother–child dyads (0.92 [95% CI − 0.81–0.97, *P* < 0.001]). Furthermore, good agreement was reported for treatment anxiety (0.79 [95% CI − 0.38–0.92, *P* < 0.001]) and moderate agreement for nausea (0.70 [95% CI − 0.38–0.87, *P* < 0.001]) and worry (0.62 [95% CI − 0.25–0.84, *P* = 0.002]). Notably, the domain communication is by far the one with the least agreement between children and mothers as well fathers at all assessment time points (Table 3 and Supplementary Fig. 3).

**Table 3:** Agreement between parent and child dyads on PedsQL 3.0 Cancer Module

	n	Mother and child reports		n	Father and child reports	
		ICC	95% CI		ICC	95% CI
*Time point 0* Pain and Hurt Nausea Procedural Anxiety Treatment Anxiety Worry Cognitive Problems Perceived Physical Appearance Communication Total	32	0.30* 0.42** 0.71*** 0.75*** 0.54*** 0.22 0.42** − 0.05 0.46**	− 0.06–0.58 0.08–0.66 0.48–0.85 0.54–0.87 0.25–0.74 − 0.12–0.52 0.09–0.63 − 0.24–0.20 0.15–0.69	19	0.57** 0.23 0.70*** 0.23 0.27 0.02 0.70*** − 0.15 0.12	0.18–0.81 − 0.26–0.62 0.38–0.87 − 0.27–0.61 − 0.23–0.64 − 0.46–0.47 0.38–0.87 − 0.42–0.24 − 0.32–0.53
*Time point 1* Pain and Hurt Nausea Procedural Anxiety Treatment Anxiety Worry Cognitive Problems Perceived Physical Appearance Communication Total	23	0.58*** 0.74*** 0.49** 0.62*** 0.77*** 0.40* 0.28 − 0.13 0.35**	0.21–0.80 0.48–0.88 0.10–0.75 0.29–0.82 0.52–0.90 0.02–0.69 − 0.11–0.61 − 0.33–0.19 − 0.07–0.66	13	− 0.07 0.34 − 0.02 0.12 0.12 0.15 0.09 − 0.06 0.08	− 0.43–0.42 − 0.15–0.72 − 0.61–0.54 − 0.37–0.59 − 0.19–0.53 − 0.41–0.64 − 0.21–0.49 − 0.14–0.20 − 0.13–0.42
*Time point 2* Pain and Hurt Nausea Procedural Anxiety Treatment Anxiety Worry Cognitive Problems Perceived Physical Appearance Communication Total	19	0.46** 0.70*** 0.92*** 0.79*** 0.62** 0.21 − 0.03 − 0.03 0.51***	0.03–0.75 0.38–0.87 0.81–0.97 0.38–0.92 0.25–0.84 − 0.12–0.55 − 0.37–0.37 − 0.15–0.19 − 0.10–0.84	10	0.38 0.51* 0.56* − 0.13 0.18 0.22 − 0.01 − 0.28 − 0.09	− 0.20–0.79 − 0.05–0.84 0.00–0.86 − 0.74–0.54 − 0.27–0.66 − 0.69–0.81 − 0.42–0.53 − 0.48–0.32 − 0.40–0.42

ICC Intraclass correlation coefficient (two-way mixed model. Absolute agreement, CI 95% confidence interval

*p ≤ 0.05; **p ≤ 0.01; ***p ≤ 0.001

Intraclass correlation coefficients reference values: ICC < 0.5: poor agreement, ICC between 0.5 and < 0.75: moderate agreement, ICC between 0.75 and < 0.90: good agreement, ICC > 0.90: excellent agreement [33]. Bold indicates significant values with at least moderate agreement

## Discussion

Caregiver proxy reports are often required as an alternative to child self-reports as the integration of PROM is more challenging in pediatric oncology. This stands in strong contrast to the evidence that patients themselves are the best reporters and that caregiver proxy reports are affected by several factors such as child’s age, sex, parental educational level, social demographics, cultural background, parent’s own HRQOL, and distress [12–14, 34–36]. Thus, there is a strong recommendation that children with cancer should be the primary reporters of their symptoms and if they are unable to provide self-reports caregiver proxy reports should be used [12]. In addition, an overall lack of fathers as caregiver proxy reporters is noted, and only a limited number of research studies have included both parents but did not separately investigate their perspectives on child’s HRQOL during active cancer therapy [12, 20, 22, 23]. One recently published study compared 120 paternal and maternal proxy reports concerning agreement on child HRQOL, but the vast majority of children with cancer were post-treatment (mean time since diagnosis 3.3 (± 1.4) years and 87% of the patients had completed therapy). The reported study design might lead to the assumption that paternal and maternal reports are interchangeable [27]. Interestingly, agreement between mother–child and father–child dyads were more likely to differ if their child was still in active treatment. However, the authors noted that this finding should be interpreted with caution as only few children were in active treatment [27]. Our study instead shows that during active cancer therapy, mother and father proxy reports differ in the level to which they agree with children’s self-reports. Namely, mothers' proxy reports, as compared to fathers' reports, showed better agreement with the children’s reports.

Children consistently reported fewer impairments than their parents, particularly one month following cancer therapy. This seems to be the main reason for disagreement between patients and parents and is in accordance with other studies showing that caregivers have the tendency to overestimate their children’s HRQOL impairments [20, 23, 37–39]. The PedsQL Generic for general HRQOL showed mainly moderate and good agreement shortly after diagnosis and agreement remained only on the physical domain with ongoing therapy. It could be assumed that this domain is the easiest one for evaluation, since inpatient stay and medical treatment are connected with observable physical restriction and impairment [24, 40]. The cancer-specific PedsQL is more informative and provides a comprehensive overview. These questions consider the fact that the daily routine care and, thus, closer contact between primary caregiver (mostly mothers) and child revealed many domains with moderate and good agreement for mother–child dyads throughout all assessment points. Interestingly, fathers reported more impairment of child’s HRQOL than mothers on nearly all domains and time points, thus, leading to a low number of domains with agreement. This might be influenced by their role as second caregiver who did not spend as much time with the child as the mothers did.

The strong disagreement between both parents and children in the domain communication of the PedsQL Cancer was highly surprising. The reason for the disagreement was that parents reported their children did not provide information about health status and did not ask questions of the health-care team. This stands in strong contrast to the child self-reports and the impressions of the health-care team. Ad hoc follow-up interviews with parents revealed that they misunderstood the question, i.e., by "no problems" they meant "always/often," which is the inverse sense of meaning. However, other studies using PedsQL did not notice such disagreement between children and proxies [22, 41, 42].

## Strengths and limitations

The main strength of this study is the approach to include the perspectives of both parents on the child’s HRQOL in a cohort of pediatric patients with diverse cancers. Moreover, the longitudinal monthly assessment during the intensive first therapy months provides an overview of the development of the parent’s perspectives, particularly the disagreement for father–child dyads assessed by the PedsQL Cancer Module. Notably, this study was performed during repetitive coronavirus disease outbreaks, which might have had negative effects on the cancer care management such as restricted visits (e.g., one single caregiver was allowed), increased fear of infection and reduced psychosocial support of patients (e.g., no service of clown doctors during lockdown). Further limitations are the monocentric study design, which includes a small sample size and restricts the description of the study group and comparison of the characteristics between patients and caregivers (e.g., cancer type, age). Since we routinely collect PRO data, we will be able to conduct more subgroup and in-depth analyses of our present results in the future. Finally, we have not collected data on the educational age of the children and, thus, were not able to analyze differences in patient–observer differences based on the children’s chronological and educational age. Despite these limitations, our findings take the first steps to characterize the perspectives of both parents on child’s HRQOL.

## Conclusions

So far, almost all studies came to the conclusion that both patient and observer assessment of HRQOL are of key value in pediatric oncology and their use is recommended by regulatory agencies and experts in the field [20, 43–45]. Nevertheless, no comprehensive guidance on how to deal with the well-known discrepancies between the child’s self-assessment and parent observer ratings in clinical studies is yet available. A clear guidance should offer recommendations in which cases to use patient and/or proxy ratings, present data on potential confounders for parental proxy ratings and differences in patient proxy accordance across the different age-groups, and offer recommendations on statistical approaches to overcome the bias. This would not only facilitate the use of PROs as endpoints in pediatric oncology, but also help to evolve the quality of assessed data in the field. The aim of this study was to add to the growing body of literature and, thus, to facilitate overarching recommendations on the patient–observer dilemma.

## Supplementary Information

Below is the link to the electronic supplementary material.

Supplementary file1 (DOCX 191 KB)
