# Supplementary material for: Disagreement between mothers' and fathers' rating of health-related quality of life in children with cancer
Source: Qual Life Res. 2023 Jan 12;32(6):1683–91. doi: 10.1007/s11136-023-03341-0 (PMC9836339; doi:10.1007/s11136-023-03341-0)
Supplement: Supplementary file 1 — Supplementary file1 (DOCX 191 KB) [file 11136_2023_3341_MOESM1_ESM.docx]

**Supplementary Figure 1**


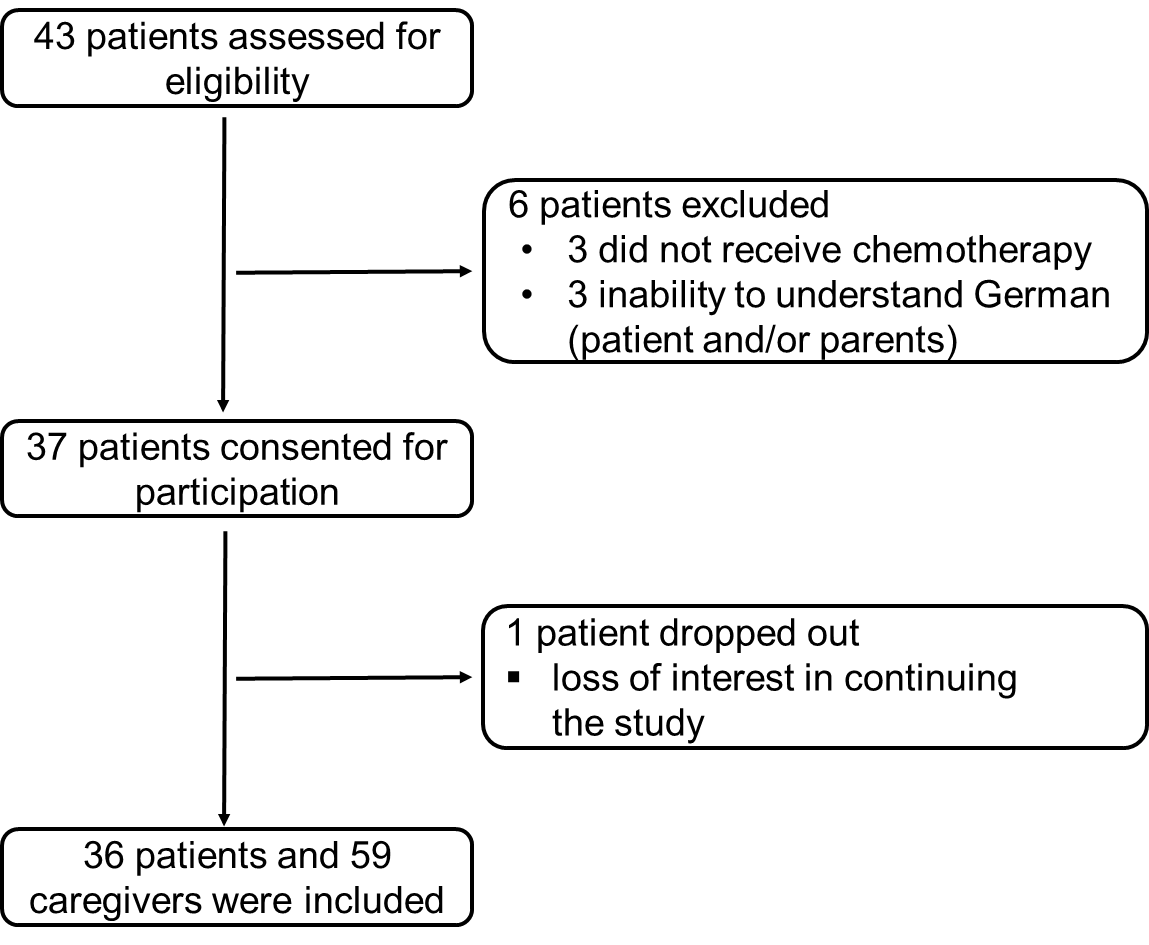


**Supplementary Figure 1: Flow chart patient enrolment.** From May 2020 to November 2021, all oncologic pediatric and adolescent patients below age 18 years were consecutively recruited. Six patients did not meet inclusion criteria as three did not receive chemotherapy and further three patients and/or parents were not able to understand German. 37 patients and their primary caregiver consented to participate. One patient dropped out in the first month. Thus, 36 patients and their 59 primary caregivers were included.

**Supplementary Figure 2**


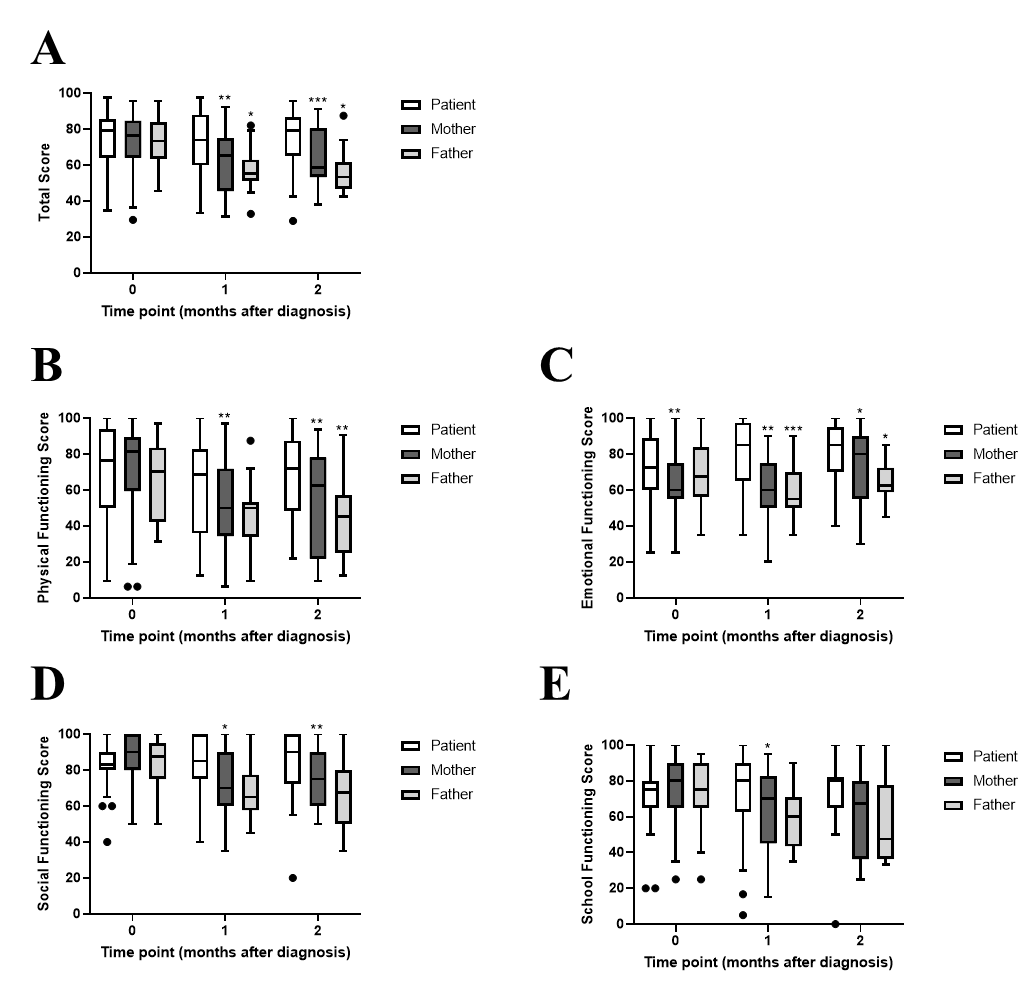


**Supplementary Figure 1: PedsQL 4.0 Generic Core Scale scores of patients, mothers and fathers.** **A** Total Score, **B** Physical Functioning Score, **C** Emotional Functioning Score, **D** Social Functioning Score, **E** School Functioning Score. P value calculated using paired t test for comparison between patients and their corresponding mothers or fathers: *p ≤ 0.05; ** p ≤0.01; *** p ≤0.001

**Supplementary Figure 2**


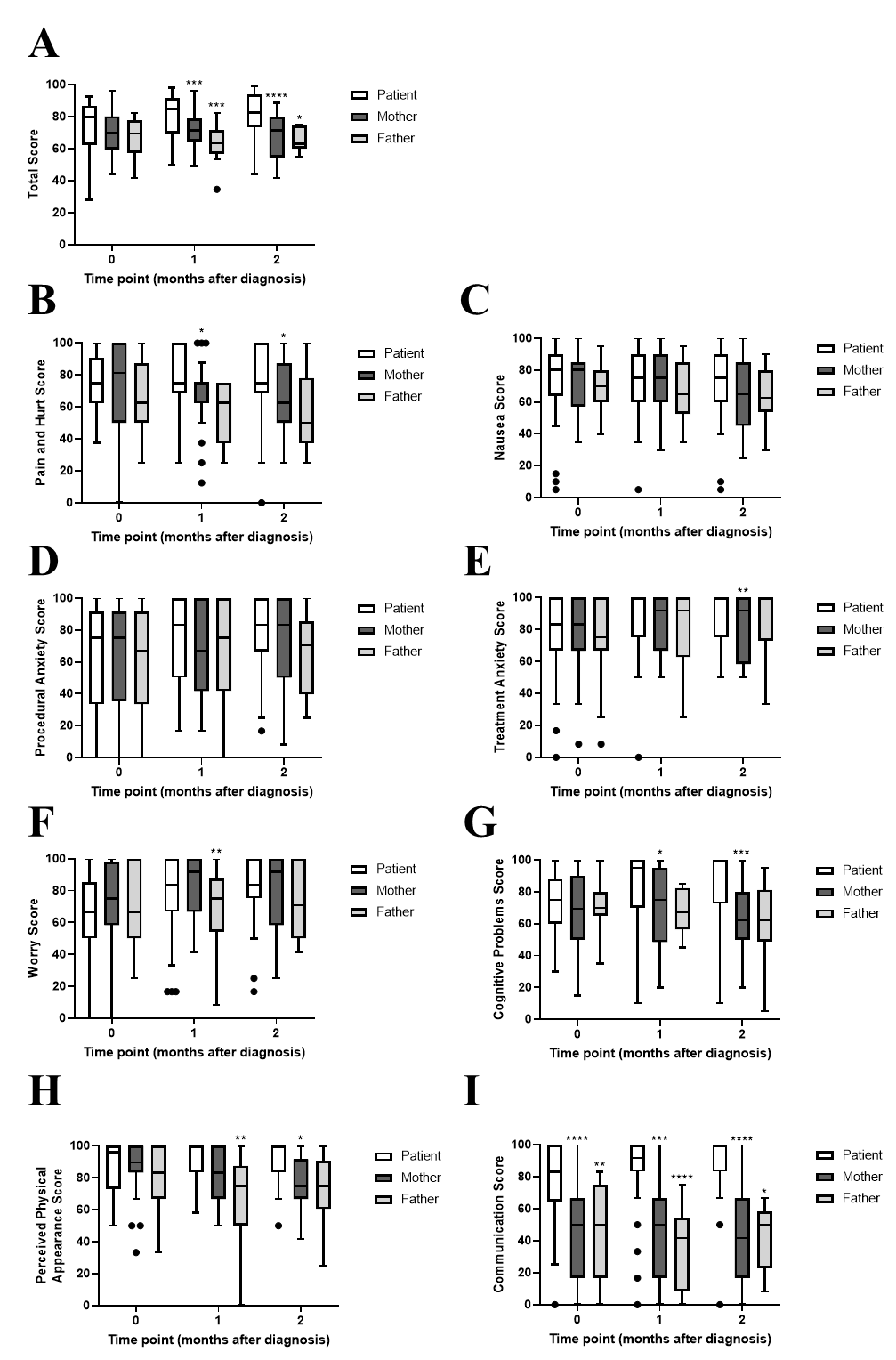


**Supplementary Figure 2: PedsQL 3.0 Cancer Module scores of patients, mothers and fathers.** **A** Total Score, **B** Pain and Hurt Score, **C** Nausea Score, **D** Procedural Anxiety Score, **E** Treatment Anxiety Score, **F** Worry Score, **G** Cognitive Problems Score, **H** Perceived Physical Appearance, **I** Communication Score. P value calculated using paired t test for comparison between patients and their corresponding mothers or fathers: *p ≤ 0.05; ** p ≤0.01; *** p ≤0.001, **** p ≤0.0001
